# Contrast enhanced computed tomography is indicative for angiogenesis pattern and display prognostic significance in breast cancer

**DOI:** 10.1186/1471-2407-14-672

**Published:** 2014-09-15

**Authors:** Jianyi Li, Yang Zhang, Wenhai Zhang, Yang Gao, Shi Jia, Jiao Guo

**Affiliations:** Department of Breast Surgery, Shengjing Hospital of China Medical University, Sanhao Street 36#, Shenyang City, Liaoning Province China

## Abstract

**Background:**

The Prognostic value of microvessel density in cancer remains unclear. Recent studies have suggested that the uneven distribution of microvessels in tumours caused the variation in sample selection which led to different prognostic outcome. The enhancement pattern of Contrast-enhanced computed tomography (CECT) is determined in part by the microvessel distribution in solid tumors. Therefore, survival analysis of tumors grouping by the enhancement pattern and the pattern of microvessel distribution is important.

**Methods:**

Survival analysis grouped by the tumor enhancement pattern and the microvessel distribution was carried out in 255 patients with invasive ductal carcinoma.

**Results:**

There were significant differences in overall survival (OS) and disease-free survival (DFS) among the homogeneous, heterogeneous and peripheral enhancement groups. There were significant differences between OS and DFS groups with uniform and uneven distributions of microvessels.

**Conclusions:**

The distribution of microvessels in a tumor is a potential prognostic indicator in patients with breast cancer, and can be assessed by CECT prior the operation.

## Background

Angiogenesis is the formation of new blood vessels from the endothelium of the existing vasculature. When a new tumor reaches 1–2 mm in size, its growth requires the induction of new blood vessels, which may lead to the development of metastases via the penetration of malignant cells into the blood circulation [1]. Microvessel density (MVD) assessment was once considered a useful indicator in the selection of those node-negative patients with breast carcinoma who are at high risk to have occult metastasis at presentation [2], and was also an commonly used important technique to quantify angiogenesis in other solid tumours [3]. However, its prognostic value remains unclear. The majority of published studies have shown a positive correlation between intratumoral MVD and prognosis in solid tumours [4], but not all studies have demonstrated such association, and this may be attributed to the significant differences in sample collection, immunostaining techniques, vessel counting and statistical analysis, although a number of biological differences may also account for the discrepancy [5]. Recently, it has been accepted that the discrepancy is due to the undifferentiated vessel density caused by variation of sample selection [6], and some researchers even began to apply computing analysis to quantify vascular properties pertaining to size, shape and spatial distributions in photographed fields of CD34 stained sections [7]. Contrast-enhanced computed tomography (CECT)-based criteria improve the diagnostic accuracy of sentinel lymph node metastases and are useful for evaluating the axillary status in patients with early-stage breast cancer [8]. The enhancement pattern of computed tomography (CT) is determined in part by the distribution of microvessels in solid tumours [9]. Therefore, it is important to carry out survival analysis grouping by the enhancement pattern and the pattern of microvessel distribution.

## Methods

### Study population

Between January 2008 and December 2011, a total of 259 patients with invasive ductal carcinoma (IDC) were treated in the Department of Breast Surgery at the Shengjing Hospital of China Medical University, Shenyang, China. Inclusion criteria for the study are: (1) no prior history of breast cancer or other malignancies, (2) no history of neoadjuvant therapy; (3) not pregnant at the time of diagnosis. All patients provided written consent for the contrast-enhanced computed tomography (CECT) scan, and agreed to undergo mammary tomography with enhancement pattern. The center and the edge of each breast cancer sample was stored in a cryogenic refrigerator (-86°C). All patients were treated with postoperational systemic adjuvant therapy (chemotherapy, radiotherapy, and endocrine therapy) guided by the National Comprehensive Cancer Network (NCCN). Follow-up examination was carried out at 4-month intervals during the first 2 years, at 6-month intervals during the next 3 years, and at 12-month intervals thereafter until December 2012. The diagnosis of local recurrence and contralateral breast cancer was supported by biopsy, and distant metastasis was diagnosed by more than two types of imaging examinations. DFS was defined as the time period from the first day after surgery to the first local recurrence or distant metastasis. OS was measured from the first day of follow-up. In this patient group, we collected anthropometric data (age at diagnosis, history of menopause, family history, surgery, chemotherapy, radiological therapy, target therapy and hormonal therapy), as well as variables related to the tumor – size, location, TNM staging, histological grade, lymphovascular invasion, metastatic nodes, estrogen receptor (ER), progesterone receptor (PgR), Ki67, P53, and microvessel density at the center and edge of tumor. Pathological tumor stage was assessed according to the criteria established by the 6th edition of the American Joint Committee on Cancer (AJCC) staging manual. The histological grade of the tumors was classified into grades I–III according to the Nottingham combined histological grade. All patients signed the Informed Consent and the study was approved by the Ethics Committee of Shengjing Hospital.

### Immunohistochemistry and fluorescence in situ hybridization

Immunohistochemistry (IHC) was performed on formalin-fixed, paraffin-embedded samples obtained from the pathology registry. Tissue sections (5-μm) were deparaffinized in xylene and rehydrated in a graded series of ethanol. Slides were treated with methanol containing 0.3% hydrogen peroxide to block any endogenous peroxidase activity. Heat-mediated antigen retrieval with the pressure cooker method was used for all staining except for the epidermal growth factor receptor (EGFR), which did not need retrieval. Antibodies recognizing the ER, PgR, and HER2 were used for immunohistochemical studies on serial tissue sections from each case; EGFR and Ki67 antibodies were used in luminal A tumours. Five markers were assessed: ER, PgR, HER2, and EGFR, which were used for breast carcinoma subtypes, and Ki67, which was used to divide luminal A tumors into two groups. The primary antibodies used in this study include ER (SP1, 1:200 dilution; ZETA), PgR (SP2, 1:200 dilution; ZETA), HER2 (CB11, 1:100 dilution; Invitrogen, Carlsbad, CA, USA), EGFR (SP9, 1:100 dilution; Invitrogen), Ki67 (K-2, 1:100 dilution; Invitrogen) and antiCD34 (class II, clone QBEnd 10, Dako-Cytomation, Glostrup, Denmark, dilution 1:50). Immunostaining was scored in a double-blinded manner by two different pathologists who were blinded to the clinicopathologic characteristics and outcome of each patient. For each antibody, the location of immunoreactivity, percentage of stained cells, and intensity were determined. The evaluation of protein expression was determined as mean ± SEM from each individual case. ER and PgR staining was assessed by Allred scoring, with positive scores ranging from 2 to 8 [10]. EGFR staining was considered positive if any cytoplasmic and/or membrane staining was observed. HER + (IHC) was defined as strong whole membrane staining in >30% of the tumor cells, and Ki67 status was expressed in terms of percentage of positive cells, with a threshold of 14% of positive cells [11]. Fluorescence in situ hybridization (FISH) analysis was performed on IHC + tumours using the PathVysion HER2 DNA Probe Kit (Vysis, Downers Grove, IL, USA). HER2-positive staining was defined as FISH-positive, and HER2-negative staining was defined as IHC 0 or negative FISH results.

### Clinicopathological subtypes

The clinical pathological subtypes of breast cancer were described, and were best matched with gene expression patterns [12]. Briefly, the subtype definitions are as follows: luminal A (ER + and/or PgR + and HER2- and/or Ki67 < 14%), luminal B (ER + and/or PgR + and HER2+ and/or Ki67 ≥ 14%), HER2 overexpression (ER-, PgR-, and HEr2+), triple-negative (ER-, PgR-, HER2-).

### Contrast-enhanced computed tomography and tumor enhancement patterns

**Figure 1:**
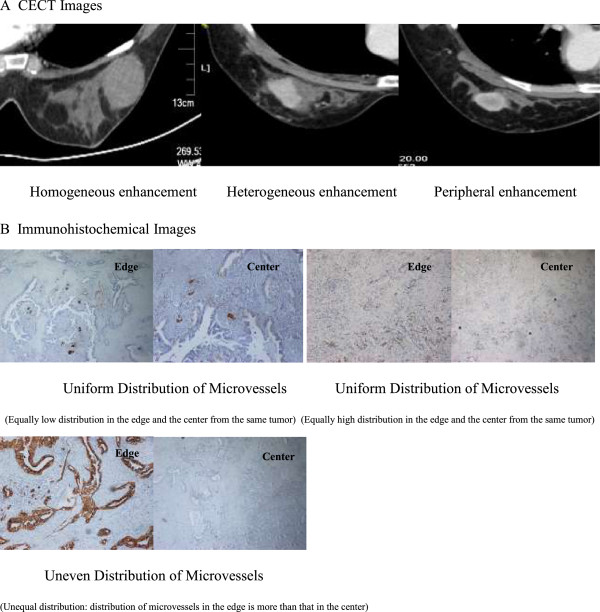
**Distribution of microvessels. A**. CECT images. **B**. Immunohistochemical images.

All CECT examinations were performed on a 64-detector row scanner (Siemens, Germany, Definition 2008 G H-SP), with the patients lying in prone position and with both arms spread out from the body. Bilateral whole breast scanning was performed within a single breath-hold with 1-mm detector raw collimation for breast cancer screening. The technical parameters were standardized as follows: 120 kV, 36 mA and 3-mm-thick contiguous section. CT images from the lower edge of breast to neck were obtained, for which 80 mL of non-ionic contrast material (Omnipaque 350, Cork, Ireland) was injected intravenously at a flow rate of 2.5 mL/s. Postcontrast CECT scanning was initiated 30 s after the start of contrast. The delay between the initiation of injection and evaluation of contrast enhancement was 60 s for early-phase imaging and 90 s for late-phase imaging. Most of the breast malignant tumor in the CECT performance had tissue fortified; only a few were not strengthened. According to CECT imaging performance morphology, the enhanced patterns of the breast tumours were classified by into peripheral enhancement, heterogeneous enhancement, homogeneous enhancement and centric enhancement [Figure 1A]. Peripheral enhancement is similar to ring strengthening, in which mainly the surrounding area of neoplasm is fortified. The CT value difference between the surrounding area and the central area is more than 10 Hounsfield units (HU). Heterogeneous enhancement means that there is an obvious difference of reinforcement in the various areas of the tissue, and the CT value difference is more than 10 HU. Homogeneous enhancement means that there is no obvious difference in reinforcement in the various areas of the tissue, and the CT value difference is less than 10 HU. In centric enhancement, mainly the central area of the neoplasm is reinforced and the CT value difference between the central area and the surrounding area is more than 10 HU. Four patients failed to enter the study for the purpose of statistical relevance, including three patients without tumor strengthened image and one patient with centric enhancement, therefore 255 patients were ultimately enrolled in this study based on the classification of peripheral, heterogeneous and homogeneous enhancement. All patients signed the Informed Consent for contrast medium hypersensitivity and the radiation dose was 9 Smv.

### Tumor samples and distribution of tumor microvessel density

**Figure 2:**
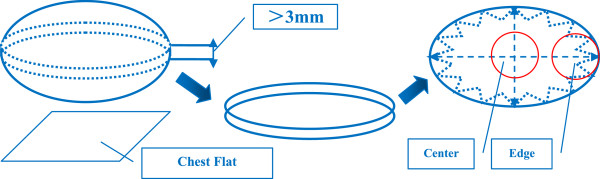
**Diagram of tumor partition.** Illustration: It was the main aim to lay the chest flat so that the edge of tumor must be located between the tumor and the normal breast tissue.

The largest section of the tumor, which was parallel to the chest wall and more than 3 mm thick, was obtained by open surgery. The center and edge of the tumor were determined by naked eye, and the weight of each specimen was more than 30 mg [Figure 2]. All samples were stored in the freezer (-86°C) after quick-freezing in liquid nitrogen. MVD was evaluated by immunohistochemical staining of tumor vessels for CD34 in whole tissue sections. Any immunopositive single cell or cluster of cells, clearly separated from adjacent clusters and from the background, with or without a lumen, was considered to be an individual vessel. Microvessels in the five most vascularized areas in a 200× magnification field (0.74 mm2) were counted simultaneously by two observers, and the average value of the five fields was calculated. The difference in MVD (DMVD) of each sample was the discrepancy from the average MVD at the edge minus that at the center of the tumor. If the discrepancy was less than 10 microvessels, the distribution of MVD was considered uniform; if the discrepancy was greater than or equal to 10, the distribution was considered uneven [Figure 1B].

### Statistics

All statistical analyses were carried out using SPSS software (version 17.0 for Windows). Grouping criteria include clinicopathological subtypes, patterns of tumor enhancement, and distribution of MVD. The correlation analyses among various groups and the various biological factors were examined by the X2 test or ANVOA analysis. For the survival analysis, Kaplan–Meier curves were constructed for overall survival (OS) and disease-free survival (DFS). The log-rank test was used to compare survival differences among the groups. Cox proportional hazards models were used to calculate relative risk accounting for covariates. P values less than 0.05 were considered statistically significant.

## Results

### Survival analysis grouping by clinicopathological subtype

**Figure 3:**
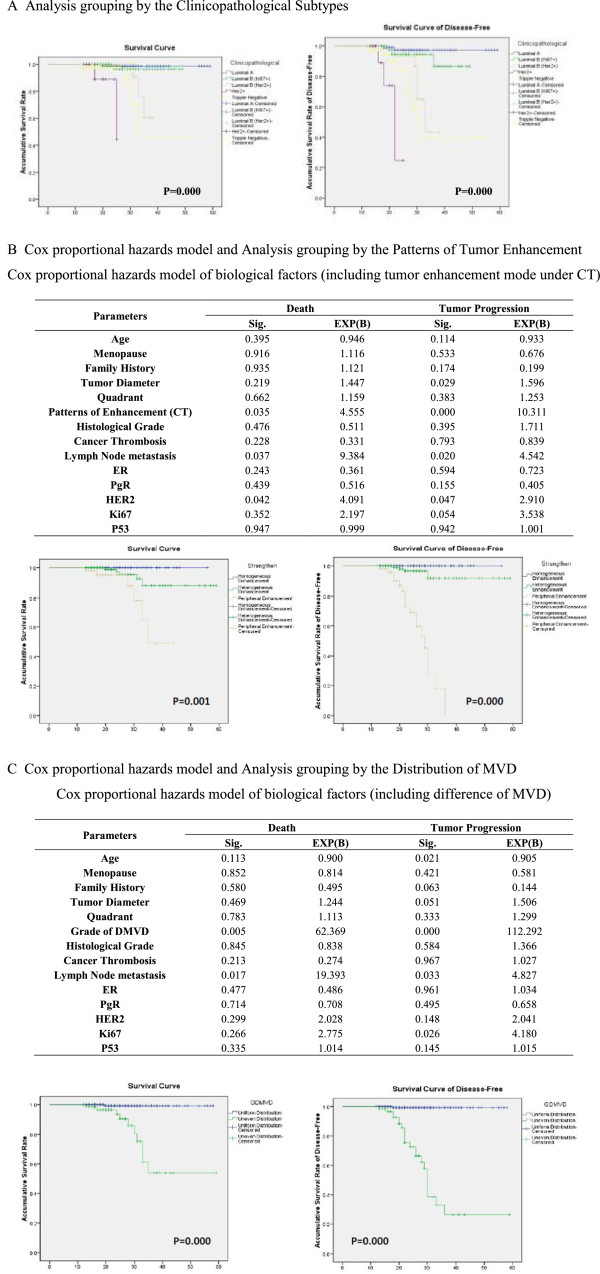
**The survival analysis and cox proportional hazards model. A**. Analysis grouping by the Clinicopathological Subtypes. **B**. Cox proportional hazards model and Analysis grouping by the Patterns of Tumor Enhancement. Cox proportional hazards model of biological factors (including tumor enhancement mode under CT). **C**. Cox proportional hazards model and Analysis grouping by the Distribution of MVD. Cox proportional hazards model of biological factors (including difference of MVD).

One hundred and nineteen patients were classified as luminal A, 52 patients as luminal B with positive Ki67, 22 patients as luminal B with HER2 over-expression, 16 patients as HER2 over-expression, and 46 as triple- negative breast cancer. The characteristics of the 5 groups are listed in Table 1. There were significant differences in tumor diameter, patterns of tumor enhancement, DMVD (edge-center), grade of DMVD, histological grades, number of metastatic nodes, clinical stage, chemotherapy program, and in targeted therapy among the groups (P < 0.05) [Table 1]. With a follow-up period of 12 to 59 months, the actual OS of luminal A, luminal B with positive Ki67, luminal B with HER2 over-expression, of HER2 over-expression, and of TNBC groups was 99.2%, 98.1%, 86.4%, 87.5%, and 91.3%, respectively, and there was significant difference among the groups (P = 0.024) [Table 1]. The median survival time of patients with luminal A, luminal B with positive Ki67, luminal B with HER2 over-expression, HER2 over-expression, and TNBC was 54, 48, 36, 29.4, and 35.2 months, respectively, and there was a significant difference among the groups (P = 0.000) [Table 1]. The actual DFS of luminal A, luminal B with positive Ki67, luminal B with HER2 over-expression, HER2 over-expression, and TNBC groups was 98.3%, 94.3%, 72.7%, 75.0%, and 82.6%, respectively, and there was a significant difference among the groups (P = 0.001) [Table 1]. The median DFS time of luminal A, luminal B with positive Ki67, luminal B with HER2 over-expression, HER2 over-expression, and TNBC groups was 54, 48, 35.4, 22.5, and 31.7 months, respectively, and there was a significant difference among the groups (P = 0.000) [Table 1]. At the same time, significant differences were observed among the curves for OS and DSF (P = 0.000; P = 0.000) [Figure 3A].

**Table 1:** **Patient characteristics and survival analysis (by clinicopathological subtype)**

Characteristic	Luminal A (n = 119)	Luminal B Ki67+ (n = 52)	Luminal B HER2 + (22)	HER2 overexpression (16)	TNBC (46)	Statistics	P
**Age (years)**	51.67 ± 10.10	50.88 ± 8.16	55.41 ± 8.98	52.13 ± 6.49	51.91 ± 10.90	0.896	0.467
**Menopause**						2.378	0.667
**Postmenopausal**	56	24	14	7	22		
**Premenopausal**	63	28	8	9	24		
**Family history**						2.075	0.722
**No**	109	49	21	16	42		
**Yes**	10	3	1	0	4		
**Diameter**	2.15 ± 0.98	2.32 ± 0.94	2.43 ± 1.07	3.09 ± 1.49	2.50 ± 1.18	3.292	0.012
**Quadrant**						16.851	0.395
**Areolar**	3	1	0	0	1		
**Inner upper**	28	8	0	2	7		
**Inner lower**	14	5	1	2	3		
**Outer lower**	21	11	3	2	6		
**Outer upper**	53	27	18	10	29		
**Enhancement patterns**						59.901	0.000
**Homogeneous**	40(33.6%)	18(34.6%)	7(31.8%)	5(31.3%)	12(26.1%)		
**Heterogeneous**	71(59.7%)	29(55.8%)	8(36.4%)	3(18.8%)	10(21.7%)		
**Peripherals**	8(6.7%)	5(9.6%)	7(31.8%)	8(50.0%)	24(52.2%)		
**Difference of MVD (Edge - Center)**	3.12 ± 6.26	3.18 ± 7.95	7.61 ± 8.53	7.98 ± 8.97	10.23 ± 8.72	9.543	0.000
**Grade of DMVD**						42.300	0.000
**Uniform distribution**	102(85.7%)	44(84.6%)	14(63.6%)	6(37.5%)	21(45.7%)		
**Uneven distribution**	17(14.3%)	8(15.4%)	8(36.4%)	10(62.5%)	25(54.3%)		
**Histological grade**						30.927	0.000
**I**	37(31.1%)	16(30.8%)	0(0%)	1(6.3%)	9(19.6%)		
**II**	77(64.7%)	29(55.8%)	16(72.7%)	10(62.5%)	34(73.9%)		
**III**	5(4.2%)	7(13.5%)	6(27.3%)	5(31.3%)	3(6.5%)		
**Cancer thrombosis**						7.806	0.099
**Negative**	89	41	11	10	34		
**Positive**	30	11	11	6	12		
**Nodal metastasis**						6.825	0.145
**Negative**	60	24	8	3	23		
**Positive**	59	28	14	13	23		
**Number of metastatic nodes**	2.09 ± 4.16	2.94 ± 5.01	6.77 ± 9.63	11.88 ± 11.37	4.89 ± 9.70	9.167	0.000
**Clinical stage**						50.721	0.000
**I**	33(27.7%)	15(28.8%)	1(4.5%)	0(0%)	10(21.7%)		
**IIA**	38(31.9%)	11(21.2%)	10(45.5%)	4(25.0%)	13(28.3%)		
**IIB**	39(32.8%)	21(40.4%)	4(18.2%)	4(25.0%)	16(34.8%)		
**IIIA**	7(5.9%)	3(5.8%)	3(13.6%)	3(18.8%)	2(4.3%)		
**IIIB**	1(0.8%)	2(3.8%)	2(9.1%)	1(6.3%)	1(2.2%)		
**IIIC**	1(0.8%)	0(0%)	2(9.1%)	4(25%)	4(8.7%)		
**IV**	0(0%)	0(0%)	0(0%)	0(0%)	0(0%)		
**P53 (%)**	27.76 ± 30.19	32.65 ± 34.17	41.55 ± 30.32	30.19 ± 32.23	35.50 ± 35.33	1.141	0.338
**Operation**						5.455	0.244
**Mastectomy**	102	47	21	16	43		
**Tumorectomy**	17	5	1	0	3		
**Chemotherapy program**						49.253	0.002
**Not performed**	1(0.8%)	0(0%)	0(0%)	0(0%)	1(2.2%)		
**CMF**	2(1.7%)	0(0%)	0(0%)	0(0%)	0(0%)		
**CAF or AC**	42(35.3%)	17(32.7%)	2(9.1%)	0(0%)	11(23.9%)		
**CEF or EC**	21(17.6%)	12(23.1%)	10(45.5%)	5(31.3%)	14(30.4%)		
**T or TC or TP**	42(35.3%)	15(28.8%)	4(18.2%)	2(12.5%)	13(28.3%)		
**TAC or A-T**	10(8.4%)	8(15.4%)	6(27.3%)	9(56.3%)	7(15.2%)		
**Radiotherapy**						6.682	0.154
**Not performed**	67	27	10	4	27		
**Performed**	52	25	12	12	19		
**Endocrine therapy**						262.436	0.000
**Not performed**	0(0%)	0(0%)	0(0%)	16(100%)	46(100%)		
**TAM**	75(63.0%)	32(61.5%)	12(54.5%)	0(0%)	0(0%)		
**LHRH**	11(9.2%)	7(13.5%)	0(0%)	0(0%)	0(0%)		
**AI**	33(27.7%)	13(25.0%)	10(45.5%)	0(0%)	0(0%)		
**Targeted therapy**						33.619	0.000
**Not performed**	119(100%)	52(100%)	20(90.9%)	13(81.3%)	46(100%)		
**Performed**	0(0%)	0(0%)	2(9.1%)	3(18.8%)	0(0%)		
**Overall survival**	99.2%	98.1%	86.4%	87.5%	91.3%	17.629	0.024
**Event**	1	1	3	2	4		
**Deaths**	1	1	3	2	3		
**Lost to follow-up**	0	0	0	0	1		
**Median survival time**	54.0	48.0	36.0	29.4	35.2	31.845	0.000
**Disease-free survival**	98.3%	94.3%	72.7%	75.0%	82.6%	56.588	0.001
**Event**	2	3	6	4	7		
**Local recurrence**	0	1	0	0	0		
**Contralateral breast cancer**	1	1	2	0	0		
**Lung metastasis**	0	0	0	1	1		
**Hepatic metastasis**	0	0	2	1	2		
**Brain metastasis**	0	0	0	1	2		
**Multi-organ**	1	1	2	1	2		
**Lost to Follow-up**	0	0	0	0	1		
**Disease-free survival**	54.0	48.0	35.4	22.5	31.7	59.248	0.000
**Follow-up time**						4.741	0.001
**Median**	23.0	25.5	30.5	17.0	20.0		
**Range**	12-59	12-49	12-38	12-25	12-53

### Cox proportional hazards model (including CT tumor enhancement patterns)

Fourteen independent biological factors were used to build a COX proportional hazard model for death and tumor progression, including the age, history of menopause, family history, tumor diameter, quadrant, patterns of CT enhancement, histological grade, cancer thrombosis, lymph node metastasis, and tumor markers. There were significant differences in the patterns of tumor enhancement, lymph node metastasis, and HER2 between death (P < 0.05) and Exp (B) (expose of the B coefficient), namely 4.555, 9.384 and 4.091, respectively. There were significant differences in the tumor diameter, patterns of tumor enhancement, lymph node metastasis, and HER2 between tumor progression (P < 0.05) and Exp (B), namely 1.596, 10.311, 4.542 and 2.910, respectively [Figure 3B].

### Survival analysis grouping by tumor enhancement patterns

Eighty-two patients were classified as homogeneous enhancement, 121 patients as heterogeneous enhancement, and 52 as peripheral enhancement. The characteristics of the groups are listed in Table 2; there were significant differences in tumor diameter, DMVD (edge-center), grade of DMVD, number of metastatic nodes, clinical stage, ER, PgR, Her2, Ki67, clinicopathological subtypes, and endocrine therapy among the groups (P < 0.05) [Table 2]. With a follow-up period of 12 to 59 months, the actual OS of the homogeneous enhancement, heterogeneous enhancement, and peripheral enhancement groups was 100%, 95.9%, and 88.5%, respectively, and there was a significant difference among the groups (P = 0.018) [Table 2]. The median survival time of the homogeneous enhancement, heterogeneous enhancement, and peripheral enhancement groups was 54, 54, and 42 months, respectively, and there was a significant difference among the groups (P = 0.014) [Table 2]. The actual DFS of the homogeneous enhancement, heterogeneous enhancement, and peripheral enhancement groups was 100%, 95.9%, and 65.4%, respectively, and there was a significant difference among the groups (P = 0.000) [Table 2]. The median DFS time of the homogeneous enhancement, heterogeneous enhancement, and peripheral enhancement groups was 54, 54, and 29.5 months, respectively, and there was a significant difference among the groups (P = 0.000) [Table 2]. At the same time, significant differences were observed among groups in the curves for OS and DFS (P = 0.001; P = 0.000) [Figure 3B].

**Table 2:** **Characteristics of patients and survival analysis (by the patterns of tumor enhancement)**

Characteristic	Homogeneous enhancement (n = 82)	Heterogeneous enhancement (121)	Peripheral enhancement (52)	Statistics	P
**Age(years)**	51.35 ± 8.51	52.24 ± 10.37	52.00 ± 9.53	0.210	0.811
**Menopause**				1.388	0.499
**Postmenopausal**	41	54	28		
**Premenopausal**	41	67	24		
**Family history**				1.095	0.579
**No**	76	111	50		
**Yes**	6	10	2		
**Diameter**	2.08 ± 0.91	2.31 ± 1.04	2.78 ± 1.28	7.028	0.001
**Quadrant**				12.629	0.125
**Areolar**	2	1	2		
**Inner upper**	13	29	3		
**Inner lower**	11	9	5		
**Outer lower**	13	22	8		
**Outer upper**	43	60	34		
**Difference of MVD (Edge - Center)**	-0.42 ± 5.72	3.73 ± 4.67	17.02 ± 3.88	211.273	0.000
**Grade of DMVD**				179.854	0.000
**Uniform distribution**	77(93.9%)	110(90.9%)	0(0%)		
**Uneven distribution**	5(6.1%)	11(9.1%)	52(100%)		
**Histological grade**				2.021	0.732
**I**	22	31	10		
**II**	51	80	35		
**III**	9	10	7		
**Cancer thrombosis**				2.910	0.233
**Negative**	60	92	33		
**Positive**	22	29	19		
**Nodal metastasis**				1.588	0.452
**Negative**	25	61	22		
**Positive**	47	60	30		
**Number of metastatic nodes**	3.80 ± 6.88	2.51 ± 5.30	6.73 ± 10.17	6.540	0.002
**Clinical stage**				19.007	0.040
**I**	20(24.4%)	32(26.4%)	7(13.5%)		
**IIA**	25(30.5%)	37(30.6%)	14(26.9%)		
**IIB**	29(35.4%)	38(31.4%)	17(32.7%)		
**IIIA**	4(4.9%)	10(8.3%)	4(7.7%)		
**IIIB**	2(2.4%)	2(1.7%)	3(5.8%)		
**IIIC**	2(2.4%)	2(1.7%)	7(13.5%)		
**IV**	0(0%)	0(0%)	0(0%)		
**ER**				31.203	0.000
**Negative**	29(35.4%)	32(26.4%)	37(71.2%)		
**Positive**	53(64.6%)	89(73.6%)	15(28.8%)		
**PgR**				34.485	0.000
**Negative**	24(29.3%)	29(24.0%)	36(69.2%)		
**Positive**	58(70.7%)	92(76.0%)	16(30.8%)		
**HER2**				11.200	0.004
**Negative**	70(85.4%)	110(90.9%)	37(71.2%)		
**Positive**	12(14.6%)	11(9.1%)	15(28.8%)		
**Ki67**				14.736	0.001
**Negative (<14%=**	53(64.6%)	77(63.6%)	18(34.6%)		
**Positive (>14%)**	29(35.4%)	44(36.4%)	34(65.4%)		
**P53 (%)**	33.98 ± 32.59	30.35 ± 31.32	30.27 ± 33.83	0.357	0.700
**Clinicopathological subtypes**				59.901	0.000
**Luminal A**	40(48.8%)	71(58.7%)	8(15.4%)		
**Luminal B (Ki67+)**	18(22.0%)	29(24.0%)	5(9.6%)		
**Luminal B (HER2+)**	7(8.5%)	8(6.6%)	7(13.5%)		
**HER2 overexpression**	5(6.1%)	3(2.5%)	8(15.4%)		
**TNBC**	12(14.6%)	10(8.3%)	24(46.2%)		
**Operation**				4.885	0.087
**Mastectomy**	72	106	51		
**Tumorectomy**	10	15	1		
**Chemotherapy program**				14.344	0.279
**Not performed**	0	1	1		
**CMF**	1	1	0		
**CAF or AC**	23	42	7		
**CEF or EC**	19	27	16		
**T or TC or TP**	27	34	15		
**TAC or A-T**	12	16	13		
**Radiotherapy**				1.560	0.458
**Not performed**	40	69	26		
**Performed**	42	52	26		
**Endocrine therapy**				53.381	0.000
**Not performed**	17(20.7%)	13(10.7%)	32(61.5%)		
**TAM**	38(46.3%)	68(56.2%)	13(25.0%)		
**LHRH**	7(8.5%)	8(6.6%)	3(5.8%)		
**AI**	20(24.4%)	32(26.4%)	4(7.7%)		
**Targeted therapy**				2.329	0.312
**Not performed**	79	119	52		
**Performed**	3	2	0		
**Overall survival**	100%	95.9%	88.5%	11.876	0.018
**Event**	0	5	6		
**Deaths**	0	5	5		
**Lost to follow-up**	0	0	1		
**Median survival time**	54.0	54.0	42.0	8.525	0.014
**Disease-free survival**	100%	95.9%	65.4%	63.908	0.000
**Event**	0	5	18		
**Local recurrence**	0	0	1		
**Contralateral breast cancer**	0	0	4		
**Lung metastasis**	0	0	2		
**Hepatic metastasis**	0	0	5		
**Brain metastasis**	0	1	2		
**Multi-organ**	0	4	3		
**Lost to follow-up**	0	0	1		
**Disease-free survival**	54.0	54.0	29.5	45.952	0.000
**Follow-up time**				2.967	0.053
**Median**	21.0	25.0	21.0		
**Range**	12-56	12-59	12-44		

### Cox proportional hazards model (including MVD distribution)

Fourteen independent biological factors were used to build a COX proportional hazard model for death and tumor progression, including age, history of menopause, family history, tumor diameter, quadrant, grade of DMVD, histological grade, cancer thrombosis, lymph node metastasis, and tumor markers. There were significant differences in the grade of DMVD and lymph node metastasis between death (P < 0.05) and Exp (B) (expose of the B coefficient), namely 62.369 and 19.393, respectively. There were significant differences in age, grade of DMVD, lymph node metastasis, and Ki67 between tumor progression (P < 0.05) and Exp (B), namely 0.905, 112.292, 4.827 and 4.180, respectively [Figure 3C].

### Survival analysis grouping by grade of DMVD

Distribution was classified as uniform in 187 patients and as uneven in 68 patients. The characteristics of the two groups are listed in Table 3. There were significant differences between groups in tumor diameter, patterns of tumor enhancement, DMVD (edge-center), number of metastatic nodes, clinical stage, ER, PgR, Her2, Ki67, clinicopathological subtypes, chemotherapy, and endocrine therapy (P < 0.05) [Table 3]. With a follow-up period of 12 to 59 months, the OS of the uniform distribution group was significantly longer than in the uneven distribution group (99.5% vs. 85.3%, P = 0.000) [Table 3]. The median survival time of both groups was 54 months, but there was a significant difference between the groups (P = 0.000) [Table 3]. The DFS of the uniform distribution group was significantly longer than that in the uneven distribution group (99.5% vs. 67.6%, P = 0.000) [Table 3]. The median DFS was significantly longer in the uniform distribution group than that in the uneven distribution group (54 vs. 31.9 months, P = 0.000) [Table 3]. At the same time, significant differences were observed between groups in the curves for OS and DFS (P = 0.000; P = 0.000) [Figure 3C].

**Table 3:** **Characteristics of patients and survival analysis (by grade of DMVD)**

Characteristic	Uniform distribution (DMVD < 10) (n = 187)	Uneven distribution (DMVD > 10) (68)	Statistics	P
**Age (years)**	51.86 ± 9.55	52.04 ± 9.84	-0.138	0.890
**Menopause**			0.389	0.572
**Postmenopausal**	88	35		
**Premenopausal**	99	33		
**Family history**			0.012	1.000
**No**	174	63		
**Yes**	13	5		
**Diameter**	2.21 ± 0.95	2.67 ± 1.31	-3.061	0.002
**Quadrant**			5.680	0.224
**Areolar**	3	2		
**Inner upper**	39	6		
**Inner lower**	17	8		
**Outer lower**	32	11		
**Outer upper**	96	41		
**Patterns of enhancement**			179.854	0.000
**Homogeneous**	77(41.2%)	5(7.4%)		
**Heterogeneous**	110(58.8%)	11(16.2%)		
**Peripheral**	0(0%)	52(76.5%)		
**Difference of MVD (Edge - Center)**	1.29 ± 5.03	15.60 ± 4.27	-20.873	0.000
**Histological grade**			2.105	0.349
**I**	50	13		
**II**	120	46		
**III**	17	9		
**Cancer thrombosis**			1.891	0.204
**Negative**	140	45		
**Positive**	47	23		
**Nodal metastasis**			0.174	0.777
**Negative**	88	30		
**Positive**	99	38		
**Number of metastatic nodes**	2.91 ± 5.97	6.21 ± 9.42	-3.302	0.001
**Clinical stage**			18.458	0.002
**I**	46(24.6%)	13(19.1%)		
**IIA**	60(32.1%)	16(23.5%)		
**IIB**	64(34.2%)	20(29.4%)		
**IIIA**	11(5.9%)	7(10.3%)		
**IIIB**	2(1.1%)	5(7.4%)		
**IIIC**	4(2.1%)	7(10.3%)		
**IV**	0(0%)	0(0%)		
**ER**			18.731	0.000
**Negative**	57(30.5%)	41(60.3%)		
**Positive**	130(69.5%)	27(39.7%)		
**PgR**			26.314	0.000
**Negative**	48(25.7%)	41(60.3%)		
**Positive**	139(74.3%)	27(39.7%)		
**HER2**			9.786	0.003
**Negative**	167(89.3%)	50(73.5%)		
**Positive**	20(10.7%)	18(26.5%)		
**Ki67**			10.827	0.001
**Negative (<14%)**	120(64.2%)	28(41.2%)		
**Positive (>14%)**	67(35.8%)	40(58.8%)		
**P53 (%)**	32.08 ± 31.58	29.90 ± 33.94	0.479	0.633
**Clinicopathological subtypes**			42.300	0.000
**Luminal A**	102(54.5%)	17(25.0%)		
**Luminal B (Ki67+)**	44(23.5%)	8(11.8%)		
**Luminal B (HER2+)**	14(7.5%)	8(11.8%)		
**HER2 overexpression**	6(3.2%)	10(14.7%)		
**TNBC**	21(11.2%)	25(36.8%)		
**Operation**			0.819	0.485
**Mastectomy**	166	63		
**Tumorectomy**	21	5		
**Chemotherapy program**			17.357	0.004
**Not performed**	0(0%)	2(2.9%)		
**CMF**	2(1.1%)	0(0%)		
**CAF or AC**	60(32.1%)	12(17.6%)		
**CEF or EC**	43(23.0%)	19(27.9%)		
**T or TC or TP**	59(31.6%)	17(25.0%)		
**TAC or A-T**	23(12.3%)	18(26.5%)		
**Radiotherapy**			2.012	0.160
**Not performed**	104	31		
**Performed**	83	37		
**Endocrine therapy**			38.443	0.000
**Not performed**	27(14.4%)	35(51.5%)		
**TAM**	99(52.9%)	20(29.4%)		
**LHRH**	13(7.0%)	5(7.4%)		
**AI**	48(25.7%)	8(11.8%)		
**Targeted therapy**			0.116	1.000
**Not performed**	183	67		
**Performed**	4	1		
**Overall survival**	99.5%	85.3%	24.308	0.000
**Event**	1	10		
**Deaths**	1	9		
**Lost to Follow-up**	0	1		
**Median survival time**	54.0	54.0	14.885	0.000
**Disease-free survival**	99.5%	67.6%	62.030	0.000
**Event**	1	22		
**Local recurrence**	0	1		
**Contralateral breast cancer**	0	4		
**Lung metastasis**	0	2		
**Hepatic metastasis**	0	5		
**Brain metastasis**	0	3		
**Multi-organ**	1	6		
**Lost to Follow-up**	0	1		
**Disease-free survival**	54.0	31.9	47.546	0.000
**Follow-up time**			0.384	0.701
**Median**	23.0	22.0		
**Range**	12-58	12-59		

## Discussion

According to a recent report from Morocco, TNBC, particularly the basal-like subgroup, has the poorest prognosis among the clinicopathological subtypes [13]. The HER2 over-expression subtype has an equally poor prognosis among Chinese women [14, 15]. The results of our study show that only 18.8% of patients with the HER2 over-expression subtype and only 9.1% of patients with luminal B (HER over-expression) subtype received targeted therapy. Therefore, the curves for OS and DFS in the patients with HER2 over-expression (luminal B HER2+ and HER2 OE) were similar to those of the TNBC group and lower than those of other groups (luminal A and luminal B Ki67). We found that the patterns of the tumor enhancement, lymph node metastasis and HER2 are significant relative risk factors for death and tumor progression.

CECT remains a cost-effective means to assess the status of axillary lymph nodes among patients with breast cancer despite the progress of positron emission tomography/computed tomography (PET/CT) and magnetic resonance imaging (MRI) [16, 17]. Beginning in January 2008, most surgeons in our institution gradually adopted preoperative CECT for assessment of axillary lymph nodes. However, a recent study suggests that tumor vascularity is a potential predictor of treatment outcomes in metastatic renal cell carcinoma, and that CECT is correlated significantly with microvessel density [18]. In our study, if the pattern of tumor enhancement was replaced by the grade of DMVD in the Cox model, the grade of DMVD and lymph node metastasis were significant relative risk factors for death, and age, grade of DMVD, lymph node metastasis and Ki67 were significant relative risk factors for tumor progression.

We carried out survival analysis according to the patterns of tumor enhancement, and found that the tumors with peripheral enhancement had the poorest prognosis and tumors with homogeneous enhancement had the best prognosis. We then conducted survival analysis according to the distribution of MVD, and found that tumours with blood vessels concentrating on the edge had the poorest prognosis compared to other tumours. Therefore, our findings suggest that the distribution of microvessels in breast cancer may determine the prognosis.

About a decade ago, Linder et al. demonstrated that angiogenesis in pancreatic tumours was not uniform, and that the tumor cells with more microvessels had greater proliferation capacity than those with fewer microvessels [19]. The uneven distribution of MVD is most likely the reasonable explanation for the differences in the prognostic value of MVD reported in different studies [20–22]. According to the theory of evolution, proliferation, anti-apoptosis/immortalization, angiogenesis, and metastasis are the “survival instinct” of the cancer cell when under the threat of hypoxia [23]. Angiogenesis is the key mechanism for cancer cell invasion and metastasis [24, 25]. There are many proteins participating in angiogenesis, such as hypoxia-inducing factor and vascular endothelial growth factor [26]. The results of our previous study also suggest that miR-20a and miR-20b are differentially distributed in breast cancer, while VEGF-A and HIF-1alpha mRNA have coincident distributions, and VEGF-A and HIF-1alpha proteins have uneven and opposing distributions to the miRNAs [27]. To date, we have only discovered the tip of the iceberg with regard to the mechanism of heterogeneity in tumor angiogenesis. However, we are confident that the distribution of microvessels in a tumor is a useful indicator for prognosis among the breast cancer patients, and can be assessed pre-operationally by CECT.

## Conclusions

The distribution of microvessels in a tumor is a potential prognostic indicator in patients with breast cancer, and can be assessed by preoperative by CECT.
